# Supplementary material for: CD3ζ-Mediated Signaling Protects Retinal Ganglion Cells in Glutamate Excitotoxicity of the Retina
Source: Cells. 2024 Jun 8;13(12):1006. doi: 10.3390/cells13121006 (PMC11201742; doi:10.3390/cells13121006)
Supplement: Supplementary file 1 [file cells-13-01006-s001.zip › cells-2965904-supplementary.pdf]

Supplementary Information for

CD3ζ-Mediated Signaling Protects Retinal Ganglion Cells in Glutamate Excitotoxicity of the Retina

Rui Du [1], Ping Wang [1] and Ning Tian [1,2,3,4,*]

[1] Department of Ophthalmology and Visual Science, University of Utah School of Medicine, Salt Lake City, UT 84132, USA

[2] Department of Neurobiology, University of Utah, Salt Lake City, UT 84132, USA

[3] Department of Biomedical Engineering, University of Utah, Salt Lake City, UT 84132, USA

[4] Veterans Affairs Medical Center, Salt Lake City, UT 84148, USA

* Correspondence: ning.tian@hsc.utah.edu; Tel.: +1-801-213-2852

Supplemental Figure and Legend

Supplementary Figure S1.

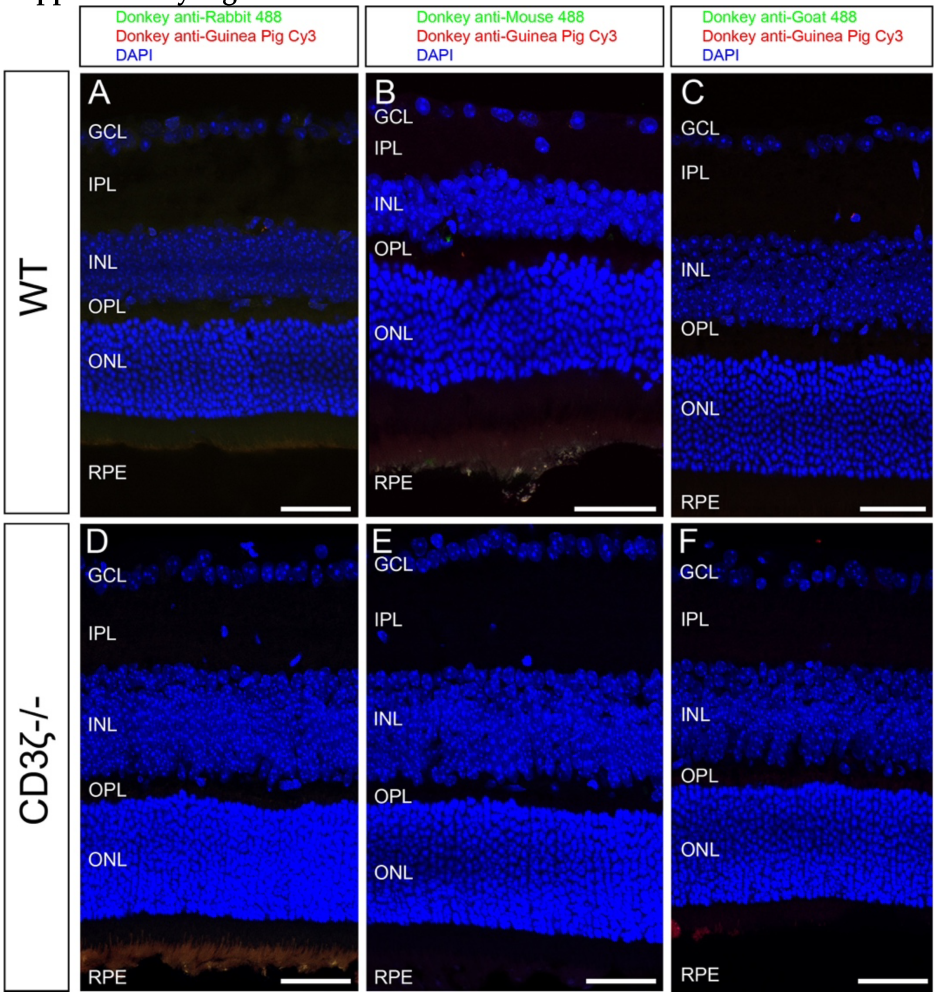

Supplementary Figure S1. Negative control immunostaining of WT and CD3ζ-/- retinal cross-sections. This figure provides negative controls for the following secondary antibodies: donkey anti-rabbit 488, donkey anti-mouse 488, donkey anti-goat 488, and donkey anti-guinea pig Cy3. (**A**) WT mouse retina co-labeled with donkey anti-rabbit 488 (green), donkey anti-guinea pig Cy3 (red), and DAPI (blue). (**B**) WT

mouse retina co-labeled with donkey anti-mouse 488 (green), donkey anti-guinea pig Cy3 (red), and DAPI (blue). (**C**) WT mouse retina was co-labeled with donkey anti-goat 488 (green), donkey anti-guinea pig Cy3 (red), and DAPI (blue). (**D**) CD3ζ-/- mouse retina co-labeled with donkey anti-rabbit 488 (green), donkey anti-guinea pig Cy3 (red), and DAPI (blue). (**E**) CD3ζ-/- mouse retina co-labeled with donkey anti-mouse 488 (green), donkey anti-guinea pig Cy3 (red), and DAPI (blue). (**F**) CD3ζ-/- mouse retina co-labeled with donkey anti-goat 488 (green), donkey anti-guinea pig Cy3 (red), and DAPI (blue). Scale bar for A-F: 40 μm.

Supplementary Figure S2.

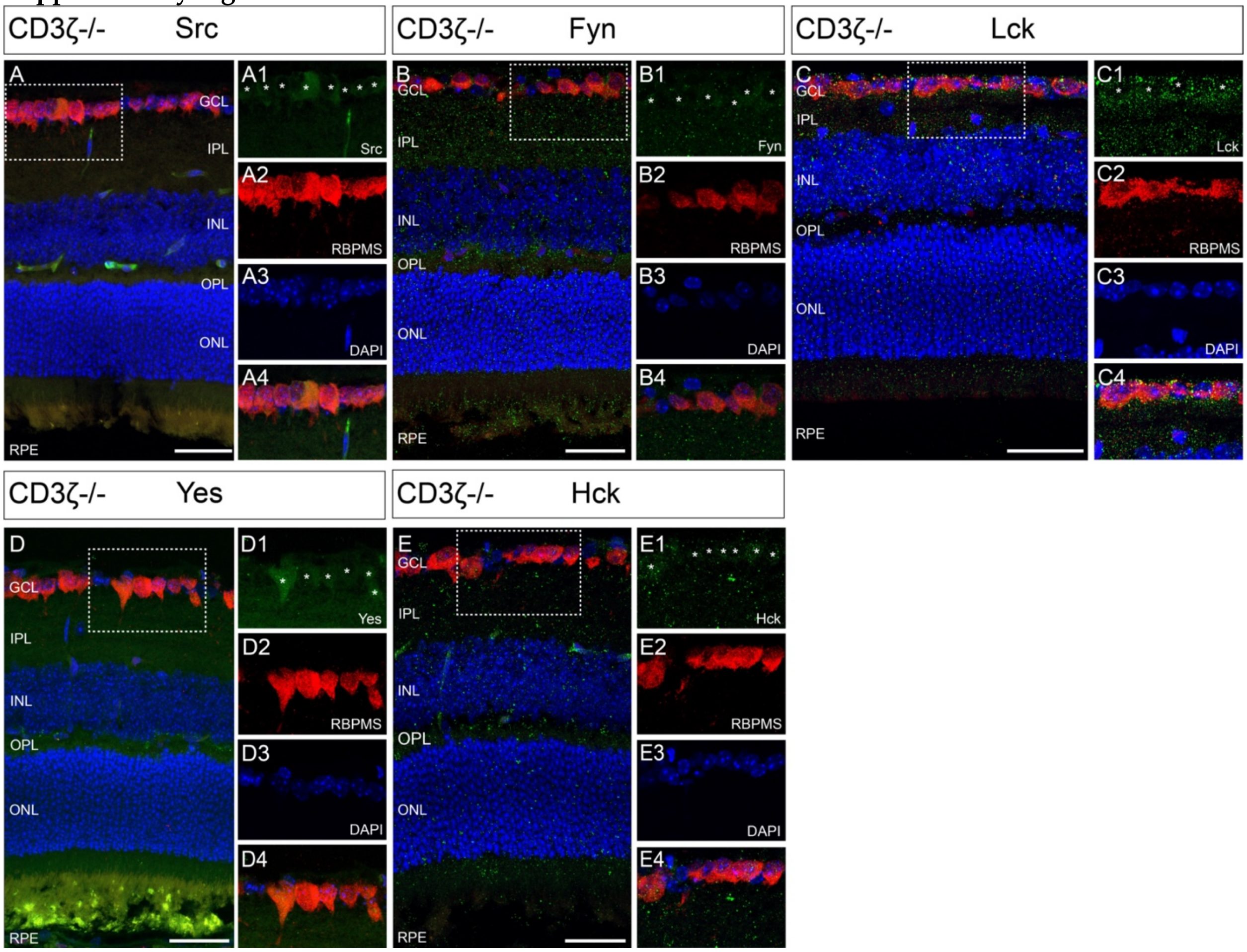

Supplementary Figure S2. Multiple SFKs are expressed by RGCs in CD3ζ-/- mouse retina. (**A**) A representative image of a retinal cross-section of a CD3ζ-/- mouse co-labeled with anti-Src antibody (green), anti-RBPMS antibody (red, for RGCs), and DAPI (blue for all nuclei in the retina section). A zoom-in view of the area in the dish-line box of panel A shows the anti-Src staining in RGCs (A1), anti-RBPMS staining (A2), DAPI staining (A3), and the overlapping of the staining of anti-Src, anti-RBPMS, and DAPI (A4). The asterisks in A1 indicate the location of RGC somas. (**B**) A retinal cross-section of a CD3ζ-/- mouse co-labeled with anti-Fyn antibody, anti-RBPMS antibody, and DAPI. B1-B4 shows the staining of anti-Fyn, anti-RBPMS, DAPI, and the overlapping of these staining of the area in the dish-line box of panel B. (**C**) A retinal cross-section of a CD3ζ-/- mouse co-labeled with anti-Lck antibody, anti-RBPMS antibody, and DAPI. C1-C4 shows the staining of anti-Lck, anti-RBPMS, DAPI, and the overlapping of these staining of the area in the dish-line box of panel C. (**D**) A retinal cross-section of a CD3ζ-/- mouse co-labeled with anti-Yes

antibody, anti-RBPMS antibody, and DAPI. D1-D4 shows the staining of anti-Lck, anti-RBPMS, DAPI, and the overlapping of these staining of the area in the dish-line box of panel D. (**E**) A retinal cross-section of a CD3ζ-/- mouse co-labeled with anti-Hck antibody, anti-RBPMS antibody, and DAPI. E1-E4 shows the staining of anti-Hck, anti-RBPMS, DAPI, and the overlapping of these staining of the area in the dish-line box of panel E. Scale bars in panels A-E: 40 μm.
